# Deficiency of *mastl*, a mitotic regulator, results in cell detachment from developing tissues of zebrafish embryos

**DOI:** 10.3389/fcell.2024.1375655

**Published:** 2024-03-11

**Authors:** Hideko Utsumi, Taijiro Yabe, Sumito Koshida, Akira Yamashita, Shinji Takada

**Affiliations:** ^1^ National Institute for Basic Biology, National Institutes of Natural Sciences, Okazaki, Aichi, Japan; ^2^ Exploratory Research Center on Life and Living Systems (ExCELLS), National Institutes of Natural Sciences, Okazaki, Aichi, Japan; ^3^ The Graduate University for Advanced Studies (SOKENDAI), Okazaki, Aichi, Japan; ^4^ Shumei University, Yachiyo, Chiba, Japan; ^5^ Graduate School of Arts and Science, The university of Tokyo, Tokyo, Japan

**Keywords:** MASTL, mitosis, zebrafish, axis elongation, chordo-neural hinge, cell detachment

## Abstract

To form tissues with unique functions and structures, it is important that the cells that comprise them maintain physical contact. On the other hand, with each mitosis, drastic changes in cell shapes, cell adhesion, and cytoskeletal architecture may cause such contacts to be temporarily weakened, risking improper development and maintenance of tissues. Despite such risks, tissues form properly during normal development. However, it is not well understood whether mitotic abnormalities affect tissue formation. Here, analysis of zebrafish embryos with aberrant mitosis shows that proper progression of mitosis is important to maintain cell contact in developing tissues. By screening mutants with abnormal trunk and tail development, we obtained a mutant with perturbed expression of some tissue-specific genes in embryonic caudal regions. The responsible gene is *mastl/gwl*, which is involved in progression of mitosis. Analysis focusing on the chordo-neural hinge (CNH), the primordium of axial tissues, shows that cell detachment from the CNH is increased in *mastl* mutant embryos. Time-lapse imaging reveals that this cell detachment occurs during mitosis. These results suggest that cells are unable to maintain contact due to abnormalities in progression of mitosis in *mastl* mutants.

## Introduction

When animal cells enter mitosis, structural changes, such as nuclear envelope breakdown, chromosome condensation, and mitotic spindle formation occur in a coordinated manner. Once microtubule attachment is accomplished, cells undergo chromosome segregation, cytokinesis, and reassembly of interphase cell structures. Accompanying these changes, mitotic cells reduce adhesiveness and become more spherical to accommodate spindle formation to ensure equal DNA separation. These changes in cell shape, cell adhesiveness, and cytoskeletal architecture constitute risks for maintenance of tissue integrity during embryogenesis. Thus, strict control of mitotic progression may reduce such risks under physiological conditions. However, it still remains to be determined whether abnormalities in progression of mitosis impair proper maintenance of cell assembly in tissues during embryogenesis.

Mitotic events proceed through a combination of regulated phosphorylation, proteolysis, and dephosphorylation. The key factor initiating mitosis is cyclin-dependent kinase 1 (Cdk1)/cyclin B, which phosphorylates many mitotic substrates and triggers chromosome condensation, nuclear envelope breakdown, and spindle formation. After reaching the spindle assembly check point (SAC), degradation of Securin triggers chromosome separation, and degradation of CyclinB inactivates Cdk1. Protein phosphatase 2A (PP2A) in complex with B55 regulatory subunits reverses Cdk1 phosphorylation, and with other phosphatases, exercises many mitotic events to reenter interphase (Hein et al., 2017; Holder et al., 2019; Lacroix et al., 2022).

To ensure phosphorylation by Cdk1/CyclinB, phosphatase PP2A-B55 should be inactivated. This is accomplished by Microtubule-associated Ser/Thr kinase-like, Mastl, also known as Greatwall (Gwl), which is activated at the outset of mitosis and antagonizes PP2A-B55. Mastl, which is a member of the AGC family of serine/threonine protein kinases, phosphorylates cAMP-regulated phosphoprotein 19 (Arpp19) and α-endosulfine (ENSA) (Gharbi-Ayachi et al., 2010; Mochida et al., 2010). Phosphorylated forms of these proteins are substrates of PP2A-B55 with very high affinity, but very slow dephosphorylation kinetics, inhibiting the action of PP2A-B55 on other substrates (Williams et al., 2014). Thus, depletion of Mastl impairs inhibition of PP2A-B55 activity, resulting in defective entry into mitosis and maintenance of mitosis in *Xenopus* egg extracts (Yu et al., 2006; Vigneron et al., 2009). Defective or delayed commencement of mitosis was also observed in RNAi-mediated knockdown of *mastl* in human cells and *Drosophila mastl* mutant (Yu et al., 2004; Burgess et al., 2010; Voets and Wolthuis, 2010). In addition to these defects, delay in transitioning to anaphase and subsequent aberrant anaphase with lagging chromosomes or DNA bridges were observed in *mastl*-defective cells (Bettencourt-Dias et al., 2004; Yu et al., 2004; Burgess et al., 2010; Voets and Wolthuis, 2010; Álvarez-Fernández et al., 2013; Diril et al., 2016). These defects are thought to be caused by incomplete phosphorylation of mitotic substrates or precocious activation of PP2A-B55, resulting in failure of chromosome condensation (Yu et al., 2004), maintenance of SAC (Diril et al., 2016), inactivation of SAC (Burgess et al., 2010), destruction of CyclinB (Voets and Wolthuis, 2015) or orderly progression of mitotic exit (Cundell et al., 2013; 2016).

By screening of zebrafish mutants involved in early embryogenesis, we identified a mutant with abnormal tail elongation. This mutant exhibited disturbances in expression of several axial or paraxial marker genes in the tail bud region. Surprisingly, we found that the gene responsible for this mutant was *mastl*. Precise analysis focusing on cells in the chordo-neural hinge (CNH), which is the origin of axial cells, revealed that abnormalities in mitotic phase progression were related to cell detachment from the CNH.

## Results

### Abnormal gene expression is observed in the posterior region of zebrafish mutant *kt441b*

**FIGURE 1:**
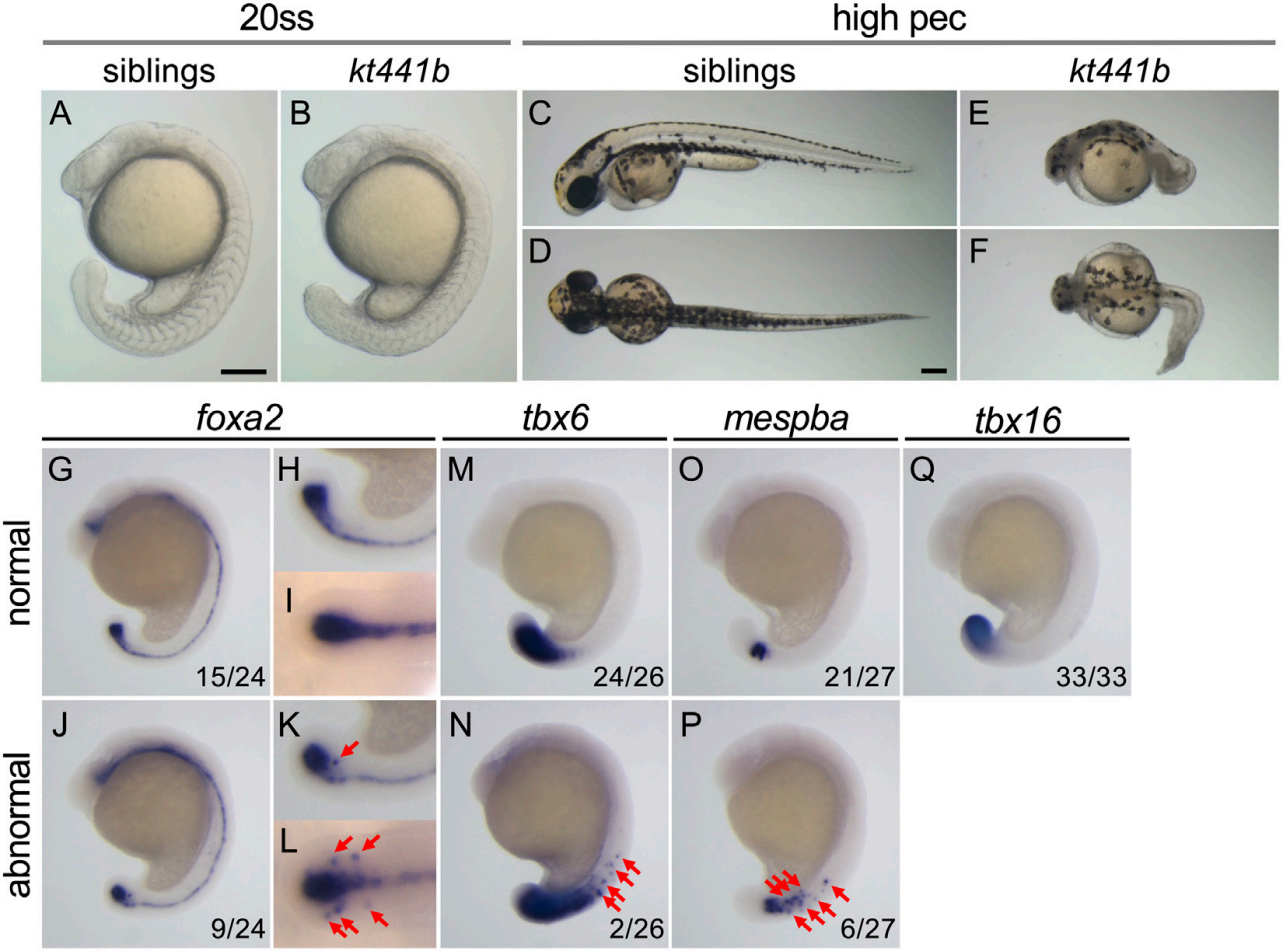
Phenotypes of *kt441b* homozygotes. **(A–F)** Morphology of siblings **(A, C, D)** and *kt441b* homozygous embryos **(B, E, F)** at the 20-somite stage; **(A, B)** and the high-pec stage **(C–F)**. Side views **(A, B, C, E)** and dorsal views **(D, F)** are indicated. Stages were estimated by morphology according to the developmental stages of zebrafish defined by Kimmel (Kimmel C. B. (1995)). Estimated high-pec embryos had been grown at 24°C for 3 days. **(G–Q)** Expression patterns of *foxa2* **(G–L)**, *tbx6* **(M, N)**, *mespba* **(O, P)**, and *tbx16* **(Q)** in embryos at the 19-somite stage generated by crossing between *kt441b* heterozygous male and female fish. Embryos showing normal gene expression patterns **(G–I, M, O, Q)** and abnormal ones are shown **(J–L, N, P)**. Side **(G, H, J, K, M–Q)** and dorsal **(I, L)** views are indicated. For *foxa2* expression, magnified images in the tailbud region are also shown **(H, I, K, L)**. In side-view images, anterior sides are located at the top. In embryos exhibiting abnormal gene expression, *foxa2, tbx6, and mespba* were additively expressed beside regions where these genes are expressed in normal embryos (red arrows). Embryos stained with *foxa2, tbx6, and mespba* probes were genotyped, and it was confirmed that penetrance of abnormal gene expression in *kt441b* homozygous embryos was 100%. Scale bars, 200 μm.

To better understand the molecular mechanism underlying axis elongation during embryogenesis, we performed ENU mutagenesis screening for mutations affecting morphogenesis of the trunk and tail in zebrafish (Koshida et al., 2005). Through this screening, we obtained a mutant in which tail elongation is defective due to a single recessive allele. In homozygotes for this mutation, *kt441b*, the tail elongation defect appeared after the 20-somite stage (Figures 1A–F). Homozygous embryos were less transparent than normal siblings, but most of them survived until the high-pec stage (42 h at 28°C, 3 days at 24°C).

To examine morphogenesis and cell differentiation in *kt441b* homozygous embryos, expression of several tissue specific marker genes, *foxa2* (the axial mesoderm, the floor plate, the hypochord, as well as their progenitors, the chordo-neural hinge (CNH); Figures 1G–L), *tbx6* (posterior presomitic mesoderm; Figures 1M, N), and *mespba* (anterior presomitic mesoderm; Figures 1O, P), *tbx16* (the tailbud; Figure 1Q) were analyzed. While no obvious difference was observed in the expression pattern of *tbx16* at the 19-somite stage among embryos obtained by crossing between *kt441b* heterozygotes*,* misexpression of *foxa2*, *tbx6,* and *mespba* was found in some embryos (Embryos with misexpression were identified by genotyping as *kt441b* homozygotes after identification of the responsible gene. Penetrance of the phenotype of misexpression was 100%). This result suggests that a defect of the gene responsible for the *kt441b* mutation causes aberrant gene expression in the axial and paraxial mesoderm in developing zebrafish embryos.

### *mastl* is the gene responsible for the *kt441b* phenotype

**FIGURE 2:**
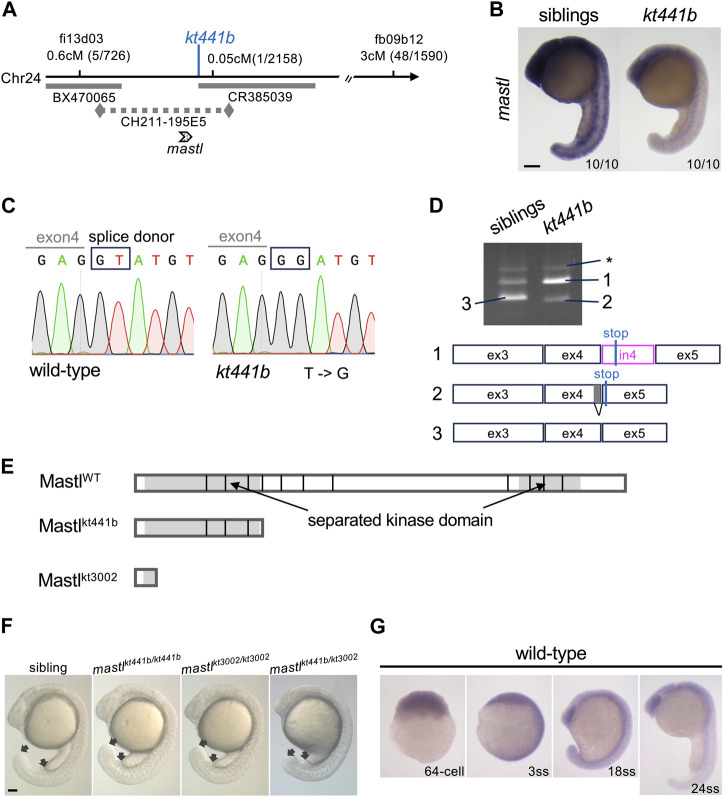
Identification of *mastl* as the gene responsible for the *kt441b* mutation. **(A)** A summary of genetic mapping of *kt441b* on chromosome 24. Numbers of recombinants and calculated distances for microsatellite markers are indicated. Gray bars indicate BAC clones, and a dotted line indicates a contig. **(B)** *In situ hybridization* showing expression of *mastl* in siblings and *kt441b* homozygous embryos at the 24-somite stage. For each, 10 embryos identified by their morphology, were stained at the same time. **(C)** Comparison of genomic sequences around a mutated nucleotide with the corresponding sequence of the wild-type allele. In *kt441b* mutation, a nucleotide in the splice donor site at the 3′ end of exon 4 of zebrafish *mastl* is changed from T to G. **(D)** PCR products of *mastl* cDNA prepared from 50 embryos of siblings and *kt441b* homozygous mutant embryos. cDNA was amplified with primers based on exons 3 and 5 of zebrafish *mastl*. Each PCR fragment from *kt441b* homozygotes was cloned and sequenced. The size of PCR fragment #3 is matched to the predicted one with proper splicing. Bands indicated with asterisks appear due to contamination of genomic DNA because they contain entire introns 3 and 4 in addition to exons 3,4 and 5 of zebrafish *mastl*. Note that siblings were a mixture of wild-type and heterozygote embryos. ex: exon, in: intron. **(E)** Schematic representation of zebrafish Mastl proteins with positions corresponding to exon-intron boundaries. Gray regions indicate the kinase domain, which is separated by a non-conserved region. One CRISPR mutant allele *mastl* ^*kt3002*^ in which 11 bp are deleted in the *mastl* gene caused frameshifts upstream of the conserved kinase domain (Supplementary Figure S1). **(F)** Morphologies of *mastl* ^*kt3002*^ homozygotes in comparison with *mastl* ^*kt441b*^ homozygotes. *mastl* ^*kt3002*^ homozygous mutants exhibited similar morphology to *mastl* ^*kt441b*^ homozygotes and did not complement *mastl* ^*kt441b*^. Arrows indicate the end of the yolk tube and the tip of the tailbud. **(G)** Expression of *mastl* mRNA at different stages of wild-type embryos. Expression of *mastl* mRNA was detectable even at the 64-cell stage, indicating that it is maternally expressed. Also, *mastl* mRNA was ubiquitously expressed from the 64-cell stage to the 24-somite stage. Scale bars, 100 μm.

To better understand the phenotype of *kt441b* mutant embryos, we next identified the gene responsible for this mutation. By linkage analysis, we mapped *kt441b* to chromosome 24. Further fine mapping revealed that *kt441b* was located between two simple sequence length polymorphism (SSLP) markers that were generated close to the EST marker fi13d03 and on the BAC clone CR385039 (Figure 2A). Because the genome sequence was not completely assembled at that time, we manually aligned contigs and BAC clones between the two SSLP markers by Blast search and examined the expression pattern of several predicted genes on the contigs. Among them, expression of *mastl* was detectable in somite stages and reduced in *kt441b* embryos (Figure 2B). We suppose that this reduction is probably due to nonsense-mediated mRNA decay (Chang et al., 2007). Sequencing of *mastl* mRNA prepared from *kt441b* homozygous embryos showed a remnant of intron 4, and genomic sequencing revealed a one-base substitution of the splice donor of intron 4 (Figure 2C). RT-PCR analysis with primers designed on exons 3 and 5 of *mastl* showed two splicing variants, both of which contain the STOP codon just downstream of exon 4. These were specifically expressed in *kt441b* homozygous embryos (Figure 2D). Both mis-spliced products encode Mastl proteins truncated around the N-terminus of the kinase domain (Figure 2E). Furthermore, by CRISPR/Cas9-mediated genome editing, we generated a mutant containing an 11-bp deletion (*mastl* ^*kt3002*^) just upstream of the conserved Mastl kinase domain (Supplementary Figure S1). This mutant showed the same live phenotype and did not complement *kt441b* (Figure 2F). Taken together, we concluded that *mastl* is the gene responsible for *kt441b* mutation.

*In situ* hybridization analysis showed that zebrafish *mastl* mRNA is expressed ubiquitously during early developmental stages, including the pre-MBT (midblastula transition) period (Figure 2G), indicating that maternally supplied *mastl* mRNA and protein can compensate for lack of zygotic products in early developmental stages (Kane et al., 1996). This is consistent with our observation that the morphological defect in *kt441b* homozygous embryos became evident after the 20-somite stage.

### *mastl* is required for proper progression of mitosis in zebrafish embryos

**FIGURE 3:**
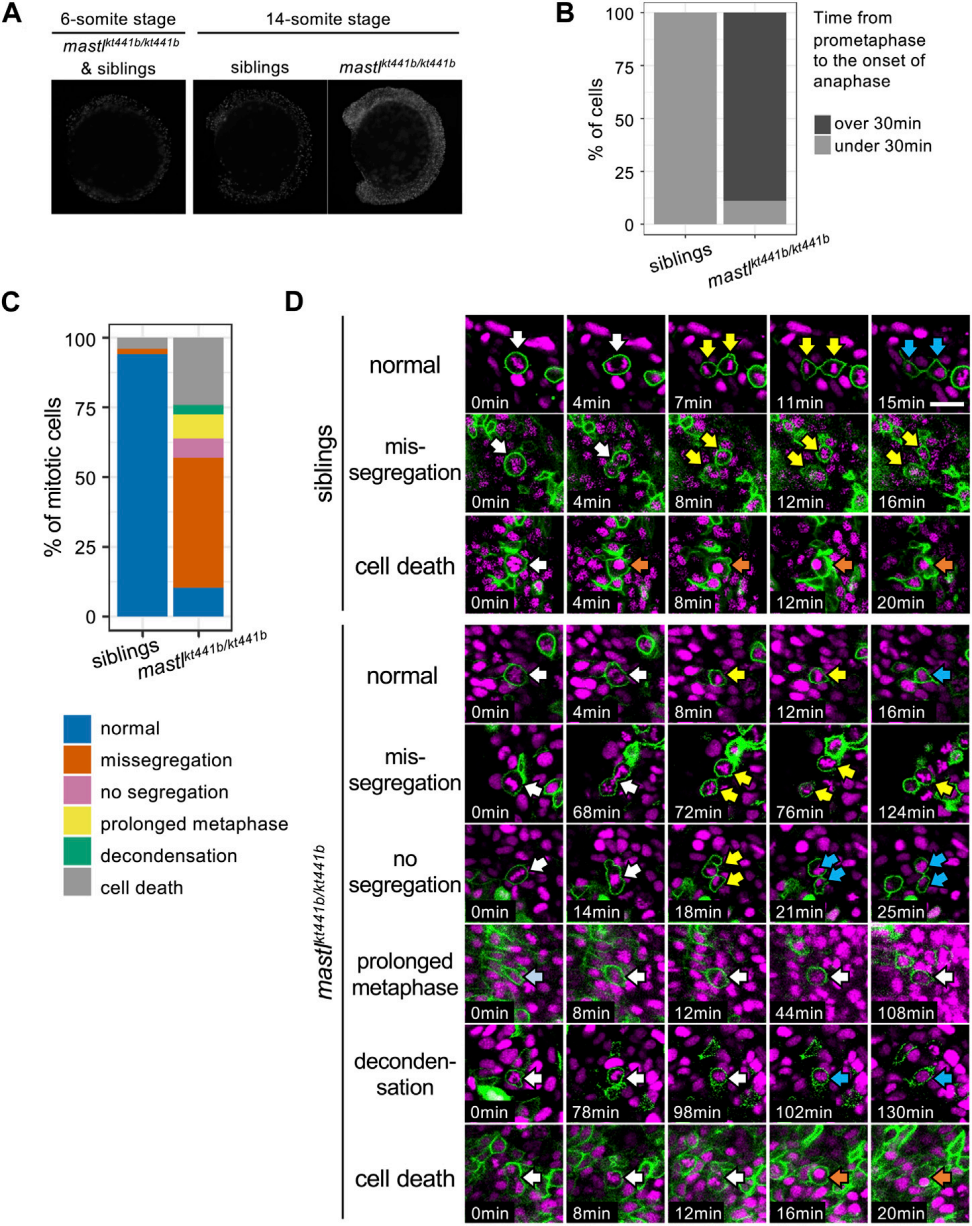
*mastl* ^*kt441b*^ homozygotes exhibit mitotic defects. **(A)** Phosphorylation of Histone H3 Ser10 (pHH3) in whole-mount embryos at the 6-somite stage (ss) and the 14 ss. At the 6 ss, no difference in phosphorylation was detectable among embryos containing siblings and *mastl* ^*kt441b*^ homozygotes. At the 14 ss, the number of pHH3-positive cells was increased in *mastl* ^*kt441b*^ homozygous embryos. For 14 ss embryos, 10 embryos of each phenotype were genotyped. **(B)** Time from prometaphase to the onset of anaphase of CNH cells. The duration was measured by time-lapse imaging of CNH cells with labeling of nuclei using Histone H2A-mChrery. The percentage of cells in which the duration of this process exceeded 30 min is indicated in dark gray. siblings: *n* = 10, *mastl* ^*kt441b*^ homozygotes: *n* = 18. **(C)** Classification of abnormalities of mitotic cells in the tail bud of siblings and *mastl* ^*kt441b*^ homozygous embryos. Embryos were labeled with Histone H2A-mCherry and sparsely labeled with membrane-Venus. All membrane-Venus labeled cells with condensed chromosomes within the first 30 min were tracked until the end of time-lapse imaging (2–3.5 h) or as long as we could track them (siblings: *n* = 50 cells, *mast* ^*kt441b*^ homozygous: *n* = 66 cells, from 3 embryos each). We classified tracked cells as follows. normal: normal cell division, missegregation: chromosomes were scattered around an elongated spindle, and cytokinesis was completed to pinch off some of the chromosomes, no segregation: cytokinesis was completed without obvious chromosome segregation, prolonged metaphase: metaphase was prolonged for more than 60 min without chromosome segregation or cytokinesis; decondensation: decondensation of chromosomes without segregation. cell death: change to hypercondensed nucleus. Time lapse images of representative cells for each type are shown in **(D)**. **(D)** Time lapse images of representative cells for each type shown in **(C)**. Green indicates membrane-Venus, while magenta indicates Histone H2A-mCherry. Mitotic cell types were classified as described in **(C)**. White arrows: mother cells with condensed chromosomes; Yellow arrows: daughter cells with condensed chromosomes; Orange arrows: hypercondensed chromosomes. Since the state of chromosome condensation was sometimes unclear, we carefully judged it referring the orthogonal views. Scale bar, 20 μm.

Mastl is a kinase that functions in mitotic progression. Thus, we examined progression of mitosis in *mastl* ^*kt441b*^ homozygous embryos. Monitoring the number of cells positive for phosphorylated Histone H3 Ser10 (pHH3), a mitotic marker, showed an increase of mitotic cells in whole bodies of *mastl* ^*kt441b*^ homozygous embryos at the 14-somite stage, but not at the 6-somite stage (Figure 3A), perhaps because of depletion of maternal products.

To see whether the increase of mitotic cells was caused by the delay of mitosis, we sparsely labeled nuclei of CNH cells by injection of Histone H2A-mCherry mRNA into one blastomere at the 8-cell stage and performed time-lapse imaging. While the average duration from prometaphase to the onset of anaphase, i.e., from the start of chromosome condensation to chromosome segregation, was 15 min (SD 2.38, n = 10, from three embryos) for cells in siblings, it was more than 84 min (SD 40.83, n = 18, from three embryos) for cells in *mastl* ^*kt441b*^ homozygous embryos. Distribution of the duration in each cell is shown in Figure 3B and Supplementary Figure S2. The average duration in cells in *mastl* ^*kt441b*^ homozygous embryos should be longer than indicated because data obtained from *mastl* ^*kt441b*^ homozygous embryos includes duration in cells that were already in prometaphase or metaphase at the start of time-lapse imaging, or those that were still in metaphase at the end of time-lapse imaging. This prolonged duration from prometaphase to the onset of anaphase is consistent with previous reports (Yu et al., 2004; Burgess et al., 2010; Voets and Wolthuis, 2010; Álvarez-Fernández et al., 2013; Diril et al., 2016).

To investigate the mitotic defect more precisely, we sparsely labeled cell membranes with Venus, along with labeling of nuclei with Histone H2A-mCherry. In this analysis, we focused on tailbud cells that entered prometaphase within the first 30 min of our time-lapse imaging and tracked them until the end of imaging, or as long as they could be tracked. Interestingly, in *mastl* ^*kt441b*^ homozygous embryos, many prometaphase cells progressed to cytokinesis after a prolonged metaphase-like state (Figures 3C, D). Two types of aberrant cytokinesis were observed. One was that chromosomes were scattered in the elongated cell, and subsequent cytokinesis was completed to pinch off some of the chromosomes (missegregation). The other type was that cytokinesis was completed without obvious chromosome segregation (no segregation). Missegregation was the major mitotic defect in *mastl* ^*kt441b*^ homozygous embryos. Additionally, in missegregated cells, after completion of cytokinesis, daughter cells often maintained condensed chromosomes for a long time, even after daughter cells were well separated. Similar defects have not been reported in previous studies of cells or organisms with impaired *mastl* function, although failure of cytokinesis and appearance of polyploid nuclei or double nuclei were observed. Prolonged condensation of chromosomes in separated daughter cells observed in *mastl* ^*kt441b*^ homozygous zebrafish embryos might be an anomaly highlighted in the context of development of tailbuds in zebrafish. Taken together, we concluded that *mastl* is required for proper progression of mitosis in zebrafish embryos, as reported in other organisms, and that cytokinesis without completion of chromosome segregation and delay of chromosome decondensation in separated daughter cells is prominent, especially in zebrafish embryos.

### Cell detachment from the CNH occurs in *mastl* ^*kt441b*^ homozygous embryos

How does perturbed gene expression occur in *mastl* ^*kt441b*^ homozygous embryos? Is it related to observed mitotic defects? To address these questions, we focused on aberrant expression of the *foxa2* gene. This is because abnormal *foxa2* expression was observed beyond CNH, which is specified at the gastrulation stage.

**FIGURE 4:**
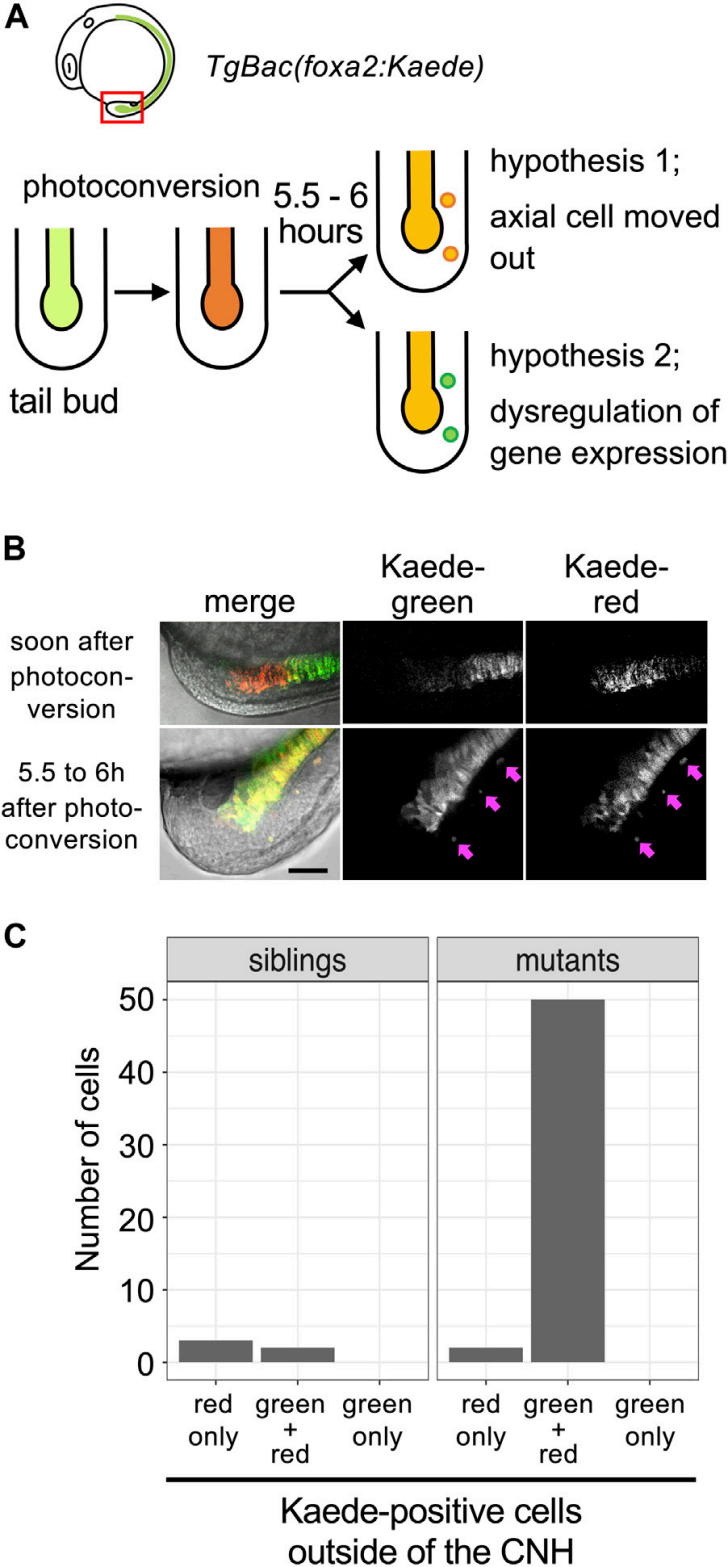
Detachment of CNH cells in *mastl* ^*kt441b*^ homozygous embryos. **(A)** The experimental design. *mastl* ^*kt441b*^ heterozygous male and female fish harboring transgene *Bac(foxa2:Kaede)* were crossed, and CNH cells of the progeny were photoconverted at the 4 to 5-somite stage. Five and half to 6 h after photoconversion, pictures of embryos were taken. Two hypotheses depicted in the figure can be distinguished by examining whether photoconverted (judged by red fluorescence) Kaede still remains in detached cells. **(B)** Examples of photoconverted tails. Pink arrows indicate Kaede-positive cells located outside the axial tissue observed at 5.5–6 h after photoconversion. Scale bar, 50 μm. **(C)** Kaede-positive cells outside of the CNH at 5.5–6 h after photoconversion were counted. The total number of cells of 3 embryos in the siblings or *mastl* ^*kt441b*^ homozygous mutant are shown.

To explain how aberrant *foxa2* gene expression occurs, we considered two hypotheses. One is that CNH cells aberrantly move out, maintaining their own gene expression profiles. The other is that cells outside of the CNH start to express *foxa2* mRNA aberrantly. To distinguish these two cases, we generated transgenic fish that express a photoconvertible fluorescent protein, Kaede, under control of the *foxa2* promoter (*TgBac(foxa2:Kaede)*). As shown in Figure 4A, Kaede expressed in CNH cells was photoconverted by UV irradiation and embryos were examined 5.5–6 h after photoconversion. Due to the stability of photoconverted Kaede protein, all cells in the CNH under irradiation should maintain the photoconverted form of Kaede, Kaede-red, throughout the experiment. Thus, if some CNH cells moved out, they should still preserve Kaede-red outside the CNH. In contrast, if cells outside the CNH newly start to express *foxa2* after UV irradiation, the unconverted form of Kaede, Kaede-green, should be expressed. Note that Kaede fluorescence of *TgBac(foxa2:Kaede)* can be detected after the 3-somite stage only in axial tissue in our experiments, even though *foxa2* mRNA starts to be expressed in the axial hypoblast and endodermal precursors from the early gastrulation stage.

All cells expressing Kaede fluorescence outside the CNH maintained Kaede-red in *mastl* homozygous embryos (*n* = 47 cells in 3 *kt441b* homozygous embryos, *n* = 5 in 11 sibling embryos; Figures 4B, C). Cells expressing only Kaede-green were not detected outside the CNH. These results support the former hypothesis, clearly showing that some CNH cells were abnormally detached in *mastl* ^*kt441b*^ homozygous embryos.

### Cells are detached from the CNH during aberrant mitosis

To examine the process of cell detachment more precisely, we next performed time-lapse imaging of *mastl* ^*kt441b*^ homozygous embryos by labeling CNH cells with Kaede fluorescent protein using *TgBac(foxa2:Kaede)*. By reverse-tracking of Kaede-positive cells located outside the CNH during time-lapse imaging, the process of detachment of these cells was examined. To make this tracking easier, nuclei were labeled sparsely by injection of Histone H2A-mCherry mRNA into one blastomere of the 8-cell stage.

**FIGURE 5:**
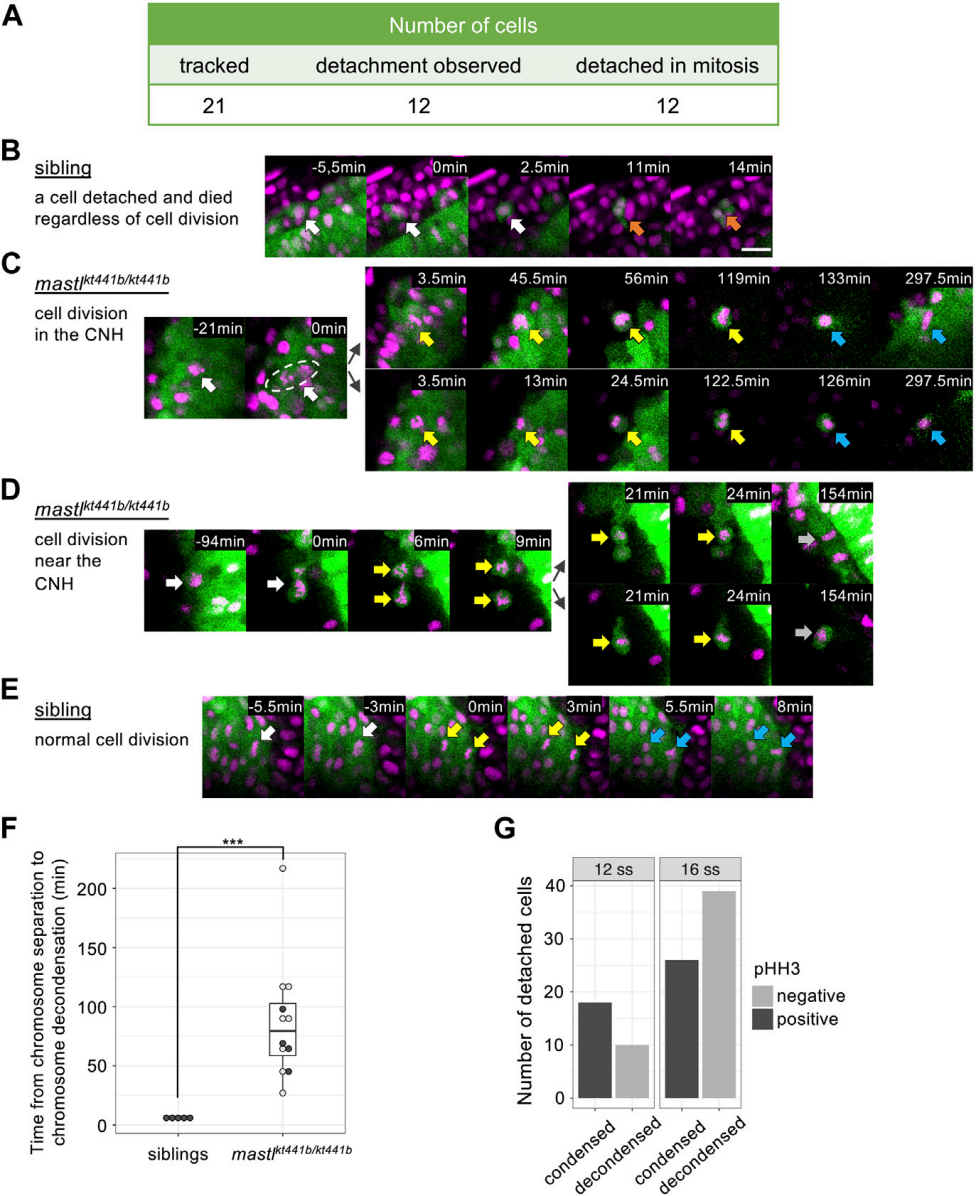
Cell detachment is related to aberrant mitosis in *mastl* ^*kt441b*^ homozygous embryos. Time-lapse imaging was carried out focusing on the tailbud region of *mastl* ^*kt441b*^ homozygous embryos carrying the *Bac(foxa2:Kaede)* transgene at the 8 to 16-somite stage. Kaede-positive cells that detached from the CNH were reverse tracked. **(A)** Summary of the tracking of detached cells in *mastl* ^*kt441b*^ homozygous embryos. Among 21 cells, 12 cells were successfully tracked back to the CNH, and all of them detached during aberrant mitosis. A schematic summary of tracked cells is shown in Supplementary Figure S3 **(B)** Time-lapse images of one detached cell in an embryo. Only one detached cell was detached among 5 sibling embryos. It was detached from the CNH at time 0 (white arrows) and died (orange arrows) soon thereafter. Chromosomes were not condensed. Scale bar, 20 μm. **(C)** A representative image of cells detaching from the CNH in *mastl* ^*kt441b*^ homozygous embryos. A mother cell in the CNH (white arrows) caused aberrant chromosome segregation at time 0. Both daughter cells detached from the CNH and maintained condensed chromosomes after segregation for around 2 h (yellow arrows). Their chromosomes appeared to decondense later (blue arrows). One of the daughter cells (upper panel) returned to the CNH. In this experiment, *mastl* ^*kt441b*^ homozygous embryos harboring the *Bac(foxa2:Kaede)* transgene were used. Nuclei were labeled by injection of Histone H2A-mCherry mRNA into 1 blastomere of the 8-cell stage. Green: CNH cells, magenta: Histone H2A-mCherry. **(D)** A representative cell that was detached and then divided near the CNH in *mastl* ^*kt441b*^ homozygous embryos. White arrows: the mother cell; Yellow arrows: condensed chromosomes; Gray arrows: chromosomes that could not be judged condensed or decondensed. **(E)** A representative image of normal cell division of CNH cells in sibling embryos. White arrows: metaphase; Yellow arrows: anaphase; Blue arrows: decondensed chromosomes in telophase. **(F)** Duration of the onset of chromosome segregation (anaphase) to chromosome decondensation. Detached cells were analyzed in *mastl* ^*kt4441b*^ homozygous embryos, comparing with CNH cells in siblings. In many cases, daughter cells in *mastl* ^*kt4441b*^ homozygotes could not be tracked until chromosome decondensation, because some daughter cells framed out, displayed reduced Kaede signals, or maintained condensed chromosomes until the time end of imaging (shown by open circles). On the other hand, examples in which time from chromosome segregation to chromosome decondensation was measured in the period of time-lapse imaging is shown by closed circles. Box plots of duration in each genotype show the first and third quartile, a line represents the median, and whiskers indicate the minimum and maximum inside 1.5 IQR (inter-quartile range). The time difference was statistically evaluated using the exact Wilcoxon-Mann-Whitney test. ***p = 0.00016 **(G)** The number of detached cells in 4 *mastl* ^*kt441b*^ homozygous embryos at the 12-somite stage and the 16-somite stage were classified by pHH3 staining and chromosome condensation. Note that all cells with condensed chromosomes were pHH3-positive.

Twenty-one cells in 3 individual *mastl* ^*kt441b*^ homozygous embryos were tracked and 12 cells were successfully tracked back to the point of detachment from the CNH (Figure 5A). The other 9 cells were already detached at the beginning of time-lapse imaging (Supplementary Figure S3). On the other hand, in 5 sibling embryos, only one detached cell, which died soon after detachment, was detected (Figure 5B, Supplementary Material S1). In *mastl* ^*kt441b*^ homozygous embryos, all 12 cells successfully traced from the CNH detached during mitosis, suggesting that cell detachment was related to mitosis. Importantly, all 12 cells exhibited aberrant chromosome segregation (Figures 5C, D, Supplementary Material S2–S6). While chromosome segregation appeared to have started, some chromosomes remained in the metaphase plane following division into two unequal clumps, which sometimes fused and then separated again. This aberrant chromosome segregation was similar to the missegregation shown in Figure 3D. These results suggests that aberrant mitosis caused by *mastl* depletion promotes cell detachment from the CNH.

**FIGURE 6:**
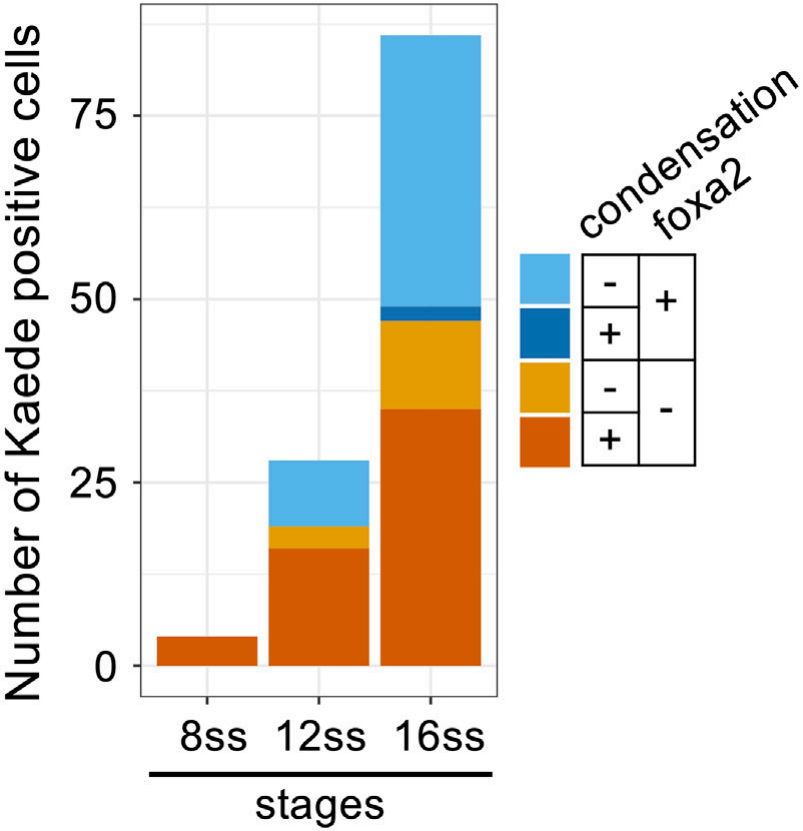
Decondensation of chromosomes and re-expression of *foxa2* in detached cells in *mastl* ^*kt441b*^ homozygous embryos. Classification of cells detached from the CNH by *foxa2* expression and chromosome condensation in *mastl* ^*kt441b*^ homozygous embryos carrying *Bac(foxa2/Kaede)* transgene at 8 ss, 12 ss and 16 ss. *In situ* hybridization of *foxa2* mRNA and immunostaining with anti-Kaede antibody was carried out. Kaede-positive cells, i.e., cells detached from the CNH, in 4 *mastl* ^*kt441b*^ homozygous embryos are classified according to *foxa2* mRNA expression and chromosome condensation. Note that most *foxa2*-expressing cells had decondensed chromosomes, suggesting that *foxa2* expression was restarted after completion of mitosis.

Interestingly, all tracked cells after “missegregation” type cell division maintained round cell shapes and condensed chromosomes for prolonged periods (Figures 5C, D, F) compared to normal cell division in siblings (Figure 5E). Because some detached cells eventually decondensed their chromosomes (Figure 5C), completion of mitosis seems to be delayed in *mastl* ^*kt441b*^ mutant embryos. We confirmed the delay of mitotic exit in detached cells by staining other *mastl* ^*kt441b*^ homozygous embryos with an antibody that specifically recognizes phosphorylation at-Ser10 of Histone H3 (pHH3); all detached cells with condensed chromosomes were pHH3 positive, while all detached cells with decondensed chromosomes were pHH3 negative, as predicted (Figure 5G; Supplementary Figure S4). Of note, double staining of *foxa2* mRNA and Kaede revealed that *foxa2* mRNA in detached cells, i.e., Kaede-positive cells, was not detected in cells with condensed chromosomes, but was mostly detected in cells with decondensed chromosomes (Figure 6A; Supplementary Figure S5). This result suggests that *foxa2* expression ceased during aberrant mitosis in detached cells, but restarted after mitotic exit. This is consistent with our observation that the proportion of *foxa2* mRNA-positive cells among detached cells, increased as development progressed.

## Discussion

Defective mitotic progression observed in zebrafish *mastl* mutant embryos is similar to that reported in other organisms in regard to prolonged time from prometaphase to the onset of anaphase, and subsequent aberrant progression of anaphase with lagging chromosomes and a DNA bridge. Additionally, we found that *mastl* mutation caused forced separation of daughter cells and a continued mitotic state, such as chromosome condensation and phosphorylation at Ser 10 of Histone H3, even after separation of daughter cells, in zebrafish embryos. Judging from the observation of aberrant chromosome separation, aneuploid cells were likely to be dominant in zebrafish *mastl* ^*kt441b*^ mutants. In contrast, multinucleate cells or polyploid cells were prominent in *mastl* knockdown of HeLa cells (Burgess et al., 2010; Voets and Wolthuis, 2010) and *mastl* conditional knockout of MEF (Álvarez-Fernández et al., 2013; Diril et al., 2016). This difference may be due to the extent of Mastl inhibition, differences in regulatory mechanisms between species, or physical pressure in tissues.

Another highlight of this study is that cells were abnormally detached from tissues in *mastl* ^*kt441b*^ mutant zebrafish embryos. In this study, we found that loss of Mastl function results in cell detachment from the CNH. Our time-lapse imaging revealed that most cell detachment occurs in mitotic phase in *mastl* mutant embryos. In mitosis, cells reorganize the cytoskeleton, stiffen the cell surface, and increase intracellular pressure to become spherical, facilitating chromosome separation and partitioning of cellular contents without being disturbed by surrounding physical forces (Taubenberger et al., 2020). In addition, cell adhesion diminished in mitosis, and at the end of mitosis, rearrangement of adhesion molecules enables two daughter cells to associate with surrounding cells (Rizzelli et al., 2020). However, in spite of the biological significance of these mitotic changes, they also seem to be a potential risk for maintenance of tissue integrity. Our results show that some abnormal cell states caused by defective mitotic progression may promote cell detachment from the CNH in zebrafish embryos. For instance, impaired reorganization of the cytoskeleton may cause abnormal mechanical force, flicking cells out of the CNH. We also assume that prolonged reduction of cell adhesion may increase the risk of detachment of CNH cells in *mastl* mutant embryos. Therefore, we speculate that cytological changes in cell morphology, adhesion and mechanical force in mitosis should be tightly coordinated in maintaining integrity of the CNH.

It is uncertain whether cytological changes in mitosis may also affect the integrity of other tissues. As far as we observed, emergence of ectopic expression differs among marker genes. In contrast to *foxa2* (a CNH marker gene), *tbx6* (a marker gene for posterior presomitic mesoderm)*,* and *mespba* (a marker gene for anterior presomitic mesoderm)*,* no ectopic expression of *tbx16* (a tailbud marker gene) was observed in *kt441b* homozygous embryos. These results suggest that the effect of prolonged mitosis on cell detachment may vary with tissue and cell differentiation status while it is also possible that the ease of restarting expression after prolonged mitosis may differ among these genes.

Details of the molecular mechanism by which *mastl* abnormalities cause prolonged mitosis and cell detachment from the CNH are currently unknown. Our results indicate that abnormalities in cell division caused by MASTL may indirectly promote cell detachment. On the other hand, it has been demonstrated that MASTL is involved in cell spreading and attachment to the ECM in mammary epithelial and breast cancer cells in a kinase-activity independent manner (Taskinen et al., 2020). MASTL induces cell contractility and cell migration by supporting expression of several proteins, including Rho guanine nucleotide exchange factor 2 (GEF-H1) and the SRF/MRTF target genes tropomyosin 4.2 (Tpm4.2), nonmuscle myosin IIB (NM-2B), and vinculin (VCL). Our results do not exclude the possibility that such abnormalities caused by defects in a kinase-independent role of *mastl* may be involved in detachment of cells from the CNH.

## Materials and methods

### Fish and embryos

Zebrafish with the TL2E background were used as the wild type, as described previously (Kishimoto et al., 2004). Zebrafish were maintained at 28 °C under a 14-h light/10-h dark cycle. Embryos were grown at 28.5 °C or 23.5 °C and their developmental stages were determined according to morphological criteria (Kimmel et al., 1995). *mastl* ^*kt441b*^ mutants were obtained with ENU-based mutant screening performed in our laboratory (Koshida et al., 2005).

Zebrafish lines generated in this study have been deposited in the National BioResource Project in Japan.

### Gene mapping

For linkage analysis, *kt441b* heterozygous fish (TL2E background) were mated with wild-type India fish to generate F1 families. Homozygous *kt441b* mutant embryos were raised from the F1 cross and selected by morphological criteria (defect of tail elongation). Pools of genomic DNA from 10 homozygotes or siblings were subjected to segregation analyses using simple sequence polymorphism (SSLP) markers of the MGH or LN54 panels. BAC or contig sequences were obtained from the Sanger Institute Zebrafish Genome Browser (http://www.ensembl.org/) and UCSC Genome Browser (https://genome.ucsc.edu/). The GENSCAN web server at MIT (http://hollywood.mit.edu/GENSCAN.html) was used for prediction of genes near the mutation point.

### Splicing valiant analysis of *mastl* mRNA expressed in *mastl* ^*kt441b*^ homozygous embryos

Total RNA was purified from 50 18-somite-stage embryos using Trizol (Thermo Fisher), and ssDNA was prepared by reverse transcription using SuperScript III (Thermo Fisher) with a *mastl* specific primer 5′-TTT​TCC​GAT​AAC​GGA​TGA​GC-3′ either from the homozygotes or their siblings. Partial fragments of *mastl* cDNA were amplified using a primer pair, 5′-GAT​GGA​GTA​TCT​GAT​TGG​AGG-3′, 5′- GAC​CAA​GAG​AGC​TGA​TCA​GTG​A-3′, designed from exons 3 and 5, respectively. Amplified PCR fragments were separated by electrophoresis and each band of *mastl* ^*kt441b*^ homozygotes was subcloned using TOPO-TA cloning (Thermo Fisher). Four clones of each band were sequenced.

### Bac transgenic zebrafish, *TgBac(foxa2:Kaede)*

To generate *foxa2*-promoter-driven Kaede transgenic fish, BAC clone DKEY-208C12, which contains the *foxa2* genomic region, was used for bacteria-mediated homologous recombination, basically following standard protocols (Bussmann and Schulte-Merker, 2011), but using *Escherichia coli* strain SW102 for recombination. Briefly, two Tol2 long terminal repeats of *Oryzias latipes* Tol2 transposon in opposing directions flanking an ampicillin resistance cassette were inserted into the BAC vector backbone. A cassette containing the Kaede open reading frame (from pKaede-S1; MBL) with an SV40 polyA signal and a reversely oriented kanamycin-resistance gene was inserted at the start ATG of the *foxa2* gene. (sequences of primers are in Supplementary Table S1). Successful recombinants were confirmed by PCR analysis. Recombinant *foxa2:Kaede* BAC was then injected with synthesized *tol2* transposase capped-mRNA into wild-type embryos at the 1-cell stage, and transgenic lines were established by screening for Kaede expression. Even though *foxa2* mRNA starts to express in axial hypoblast and endodermal precursors from the early gastrulation stage, Kaede fluorescence of the *Bac(foxa2:Kaede)* transgene can be detected after the 3-somite stage.

### CRISPR/Cas9-mediated *mastl* mutation

Construction of guide RNA vectors and preparation of single-guide RNA (sgRNA) and Cas9 mRNA were performed as described previously (Cong et al., 2013; Hwang et al., 2013). The sgRNA sequence for targeting *mastl* was as follows: 5′-TGA​AAG​CTC​CTT​CCA​TCG-3’. Mutation efficiency was assessed by performing a T7 endonuclease assay (Chen et al., 2012) with the following primers: 5′-GAC​TTA​CCA​TCA​GCC​AGT​TG’, 5′-TGT​AAC​GTT​AGC​GGA​GTA​CG-3′. Details on *mastl* mutagenesis and sequences are provided in Supplementary Figure S1. For microinjections, 1 nL of injection solution (25 ng/μL sgRNA, 0.4 μg/μL Cas9 mRNA, 0.2 M KCl and 0.05% Phenol Red) was injected into one-cell-stage zebrafish embryos using an IM300 micro injector (Narishige).

### Genotyping primers

For genotyping of *mastl* ^*kt441b*^ embryos, genomic DNA fragments amplified by PCR using mastl-genoF: 5′- TGA​TTT​GGA​GTG​TTT​TGT​TTG​TC-3′ and mastl-genoR: 5′- TAT​TAA​TTA​TTA​GGA​AAT​AGA​AAG​AT-3′ were digested with MboI, which can digest DNA fragment from *mastl* ^*kt441b*^, but not from *mastl* ^*wt*^.

For genotyping of *mastl* ^*kt3002*^, the following primers were used to amplify each allele of genomic DNA independently. For *mastl* ^*kt3002*^: 5′-GTG​GAC​TGA​AAG​GAA​GAC​TC-3′ and 5′-AGC​ACG​AAG​TCC​TCG​CTT​C-3′, for *mastl* ^*wt*^: 5′-AAC​AGT​GCA​GTG​AAA​GCT​CC -3′ and 5′- AAC​GTT​AGC​GGA​GTA​CGT​AC-3’.

### Photoconversion of kaede-expressing fish

Photoconversion was carried out using the epifluorescence setup on a Zeiss Axio Imager Z1 microscope. Kaede-expressing embryos at the 6-somite stage were mounted on glass slides in 1/3 Ringer’s solution without removing the chorion. Directions of embryos were adjusted manually by moving the coverslip. A GFP filter set was first used to identify the region for photoconversion, and the size of the target field was adjusted to include the chordo-neural hinge region by adjusting the diameter of the diaphragm in the epifluorescence light path. Once target cells were in focus, a DAPI filter set was chosen, and UV light was switched on for 30 s at maximum intensity. After photoconversion, embryos were returned to the dishes with 1/3 Ringer’s solution and kept in the incubator at 24°C for 5.5–6 h. Embryos were mounted as described below and imaged on a LEICA SP8 confocal microscope.

### Time-lapse imaging and processing

Male and female *mastl* ^*kt441b/wt*^ *; TgBac*(*foxa2:Kaede*)^*+/−*^ fish were crossed and resulting embryos were labeled with Histone H2A-mCherry by injecting RNA into a blastomere at the 1-cell stage or into a blastomere at the 8-cell stage. Embryos that had Histone H2A-mCherry labeling in the tailbud region were selected for imaging. Embryos at the 6-somite stage were embedded in 0.5% low-melting-point agarose in 1/3 Ringer’s solution and imaged using a Leica SP8 confocal microscope with a 40x water immersion lens. Z-stacks were collected at 3–4 min intervals for 3–4 h. Cell movements were tracked manually, checking the orthogonal view with Las (Leica) or ImageJ. Images were compiled to movies with ImageJ.

### *In situ* hybridization

Whole-mount *in situ* hybridization was performed as previously described (Koshida et al., 2005). Briefly, dechorionated embryos were fixed with a 2-h incubation at room temperature in 4% paraformaldehyde (PFA)/PBS. After fixation, embryos were dehydrated by treatment with methanol and kept at −20 °C until use. Hybridization was performed at 65 °C and signals were detected using anti-Digoxigenin-AP (Roche; 111093274910) and BM Purple AP Substrate, precipitating (Roche; 11442074001). For simultaneous detection of Kaede fluorescent protein with detection of *foxa2* mRNA, anti-Digoxigenin-POD antibody (Roche; 11207733910) and a TSA Plus Cyanine3/Fluorescein System (Perkin Elmer; NEL753001KT) were used.

Plasmids for probes of *foxa2*, *tbx6*, and *mespba* were kindly donated by Drs. Phillip W. Ingham (Strähle et al., 1993), Masataka Nikaido (Nikaido et al., 2002), and Hiroyuki Takeda (Sawada et al., 2000). Primers for cloning zebrafish *mastl* probe were as follows: 5′-TAT​GCC​ATC​AAG​GTG​GTG​AA-3′, 5′-AGG​CCA​TGA​GGA​TGA​TCA​AG-3′

### Immunohistochemistry

Embryos were fixed with 4% paraformaldehyde (PFA)/PBS for 2 h at room temperature. Permeabilization was done with 0.5% Triton X-100 at room temperature for 1.5 h or acetone at −20°C for 5 min (for anti-phosphorylated Histone H3 Ser 10 antibody) and then incubated with 1/500 diluted rabbit anti-Kaede antibody (MBL; PM012) or 1/1000 diluted rabbit anti-phosphorylated Histone H3 Ser10 antibody (Millipore; 06–570) overnight at 4 °C. Signals were detected with 1/1,000 diluted anti-rabbit IgG Alexa 488 (Thermo Fisher; A11008) or 555 (Thermo Fisher; A21429). For simultaneous detection of Kaede and pHH3, Kaede-green fluorescence was detected without staining.

## Data Availability

The original contributions presented in the study are included in the article/Supplementary Material, further inquiries can be directed to the corresponding author.
