# Supplementary material for: Deficiency of mastl, a mitotic regulator, results in cell detachment from developing tissues of zebrafish embryos
Source: Front Cell Dev Biol. 2024 Mar 11;12:1375655. doi: 10.3389/fcell.2024.1375655 (PMC10964716; doi:10.3389/fcell.2024.1375655)
Supplement: Supplementary file 2 [file Table1.pdf]

Table S1. Primer sequences for construction to foxa2:Kaede BAC

Primer	Sequence (5' to 3')
**Primers to amplify iTol2-amp with homology arm of BAC vector bone**	
pIndigo_HA1_itol2_F	ttctctgtttttgtccgtggaatgaacaatggaagtccgagctcatcgctATCCAGATCGATCTGCGAAG
pIndigo_HA2_itol2_R	agccccgacacccgccaacacccgctgacgcgaaccccttgcggccgcatCCATCTGGCCTGTGTTTCAG
**Primers to amplify Kaede-pA-Kanamycin cassette with homology arm to foxa2 genome region**	
foxa2_HA1_Kaede_F	CTCGTGTGGGGAAGCGTTTTAAAAAGTAGACGAAACTTTTTTCCAGGATGggtctgattaaaccagaaat
foxa2_HA2_kan_R	GTGCTCCAGTCTGCTGCGTGTTCGTGTCCCTCCATTTTGACAGCACCGAGagctagaaagccacgttgtg
