# Supplementary material for: Deficiency of mastl, a mitotic regulator, results in cell detachment from developing tissues of zebrafish embryos
Source: Front Cell Dev Biol. 2024 Mar 11;12:1375655. doi: 10.3389/fcell.2024.1375655 (PMC10964716; doi:10.3389/fcell.2024.1375655)
Supplement: Supplementary file 3 [file Presentation1.pdf]

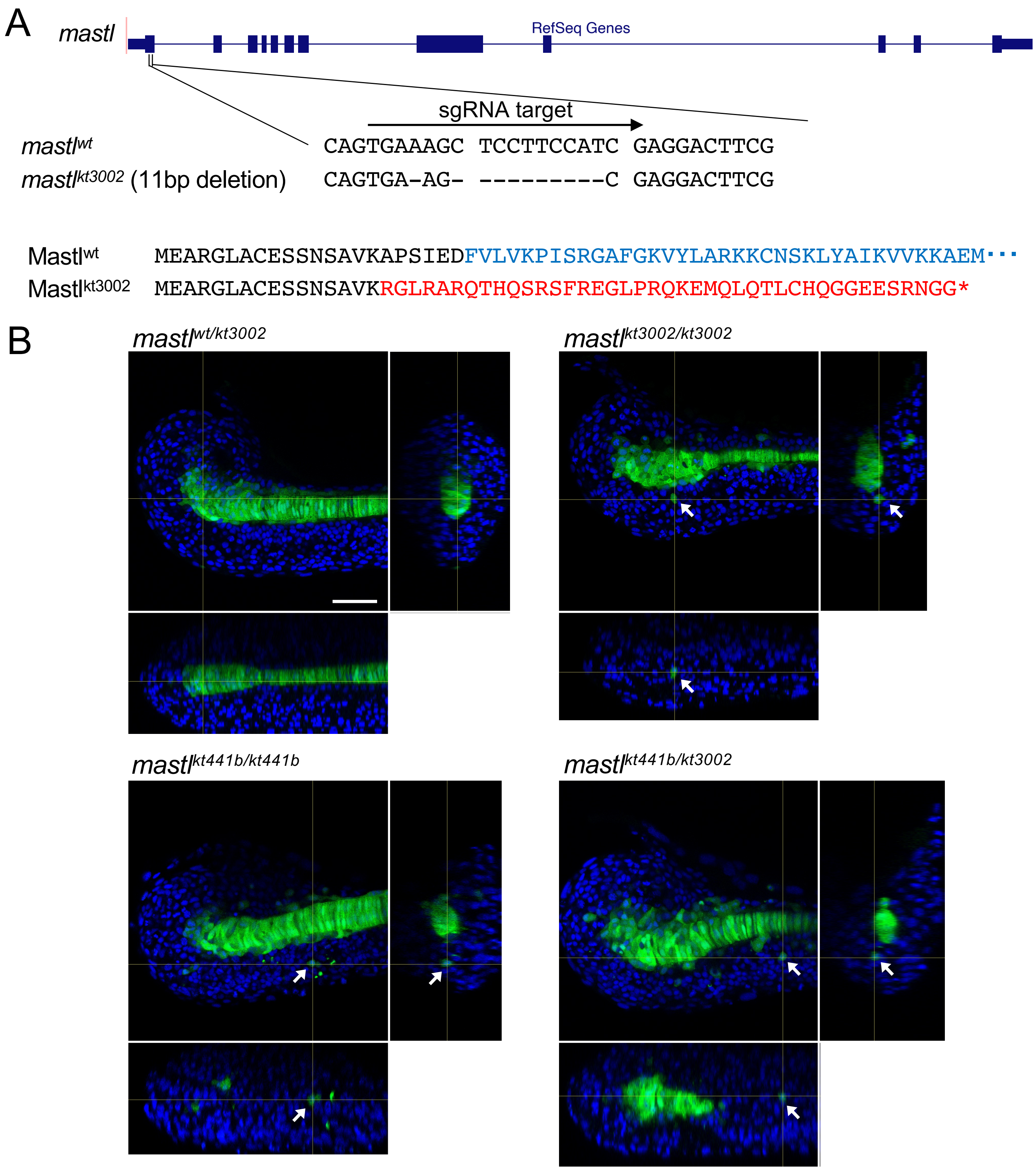

Supplementary Figure 1. CRISPR/Cas9-mediated mutagenesis of the *mastl* gene in zebrafish
(A) Generation of a *mastl* mutant allele, *mastl*^{kt3002}, by use of the CRISPR/Cas9 system. The gene structure of *mastl* in the zebrafish genome adapted from the genome browser of the University of California Santa Cruz (http://genome.ucsc.edu; Howe K, et al. 2013) is indicated at the top. Boxes show exons. In particular, coding regions are indicated by bold boxes. Upstream of the conserved kinase domain (amino acids shown in blue at the bottom) was targeted, resulting in an 11-bp deletion for *mastl*^{kt3002}, as shown in the middle. The amino acid sequence generated by this deletion is shown in red at the bottom. (B) Phenotypes of embryos carrying *mastl*^{kt3002}. Sagittal images of the tailbud regions of fixed embryos at the 16-somite stage are shown. Orthogonal XZ and YZ planes of each image are also displayed at the bottom and on the right, respectively. Genotypes of embryos are shown at the top of each orthogonal image. Since these embryos carry the *Bac(foxa2:Kaede)* transgene, CNH cells are labeled by Kaede fluorescence. Nuclei in each embryo are visualized by DAPI staining (blue). Kaede-positive cells outside the CNH are indicated by white arrows. As in the case of *mastl*^{kt441b} homozygotes, Kaede-positive cells were observed outside the CNH in *mastl*^{kt3002} homozygous embryos. *mastl*^{kt3002} does not complement *mastl*^{kt441b} for this phenotype. Scale bar, 50 µm. Howe, K. et al. (2013) "The zebrafish reference genome sequence and its relationship to the human genome," *Nature*, 496, 498–503. doi: 10.1038/nature12111

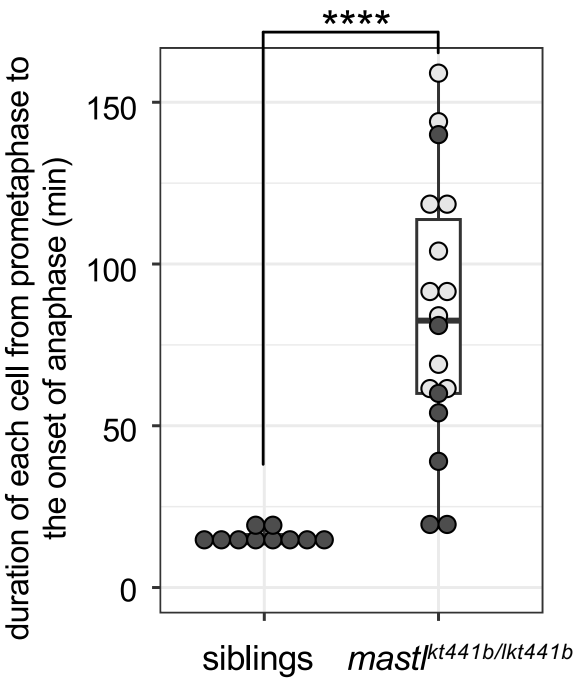

Supplementary Figure 2. Progression from prometaphase to anaphase is prolonged in *mastl*^{kt441b} homozygous embryos. The period from chromosome condensation (prometaphase) to chromosome separation (the onset of anaphase) in each CNH cell was measured by time-lapse imaging in which nuclei were sparsely labeled with Histone H2A-mCherry (n=10 from 3 sibling embryos and n=18 from 3 *mastl*^{kt441b} homozygous embryos). Because this period was too long in many cells of *mastl*^{kt441b} homozygous embryos, the onset of chromosome condensation or chromosome separation could not be confirmed during our imaging analyses (11 of 18 cases; shown by open circles). On the other hand, examples in which time from prometaphase to the onset of anaphase was measurable during the period of time-lapse imaging, are shown with closed circles. Box plots of durations in each genotype show the first and third quartile. A line represents the median, and whiskers indicate the minimum and maximum inside 1.5 IQR (inter-quartile range). The difference in duration was statistically evaluated using the exact Wilcoxon-Mann-Whitney test. ****p=6.096e-07

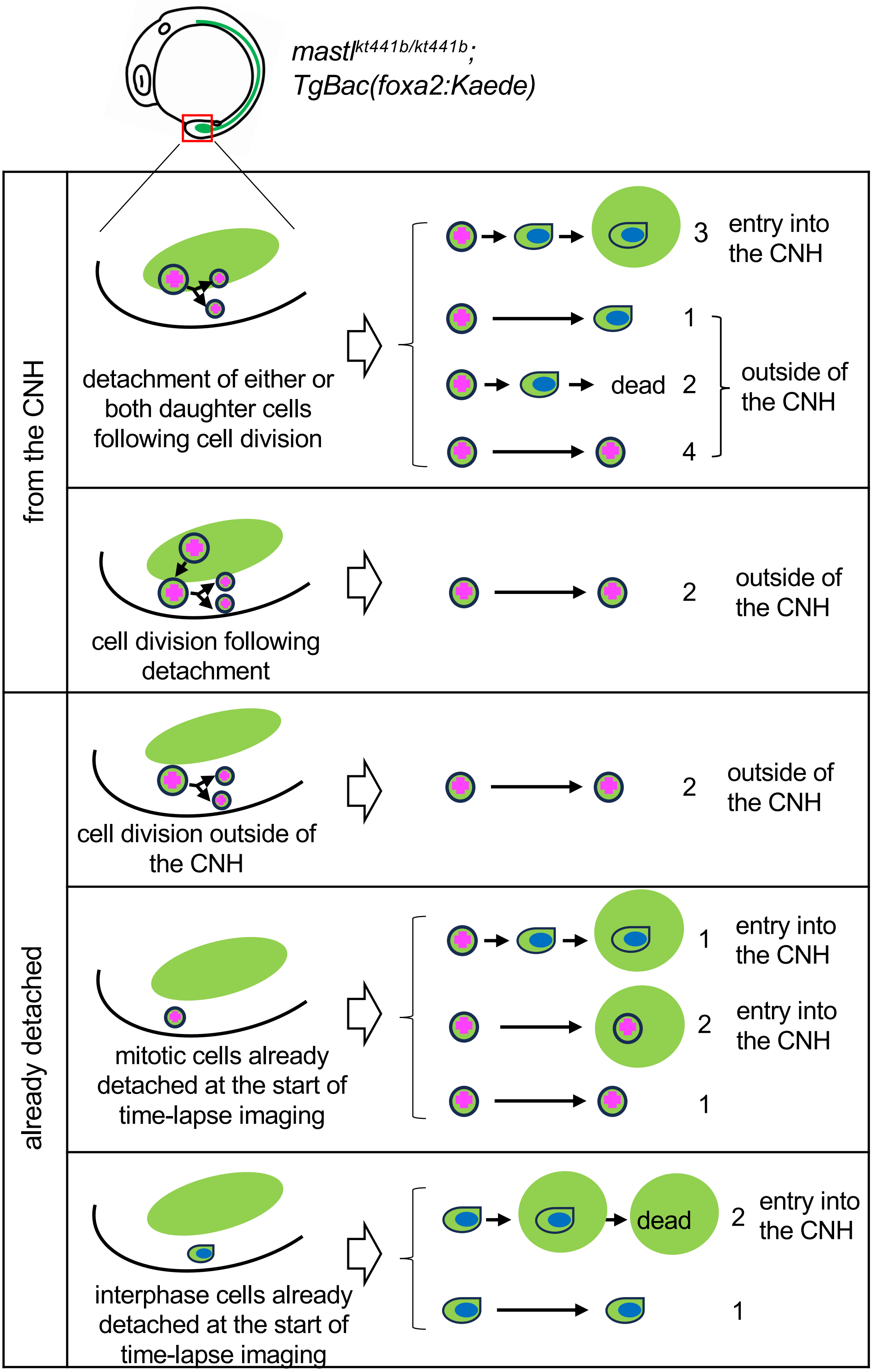

Supplementary Figure 3. Summary of tracking Kaede-positive cells outside the CNH in *mastl*^{kt441b} homozygous embryos (related to Figure 5A)

As shown in Figure 5A, CNH cells were labeled in *mastl*^{kt441b} homozygous embryos carrying the *Bac(foxa2:Kaede)* transgene. In these embryos, nuclei were sparsely labeled with Histone H2A-mCherry. Kaede- and mCherry-positive cells outside the CNH were tracked with time-lapse imaging. Numbers of daughter cells showing each behavior type are shown at the right. Pink: condensed chromosomes indicating mitotic states; Blue ellipse: interphase nuclei. Large green circles show tracked cells entered the CNH region.

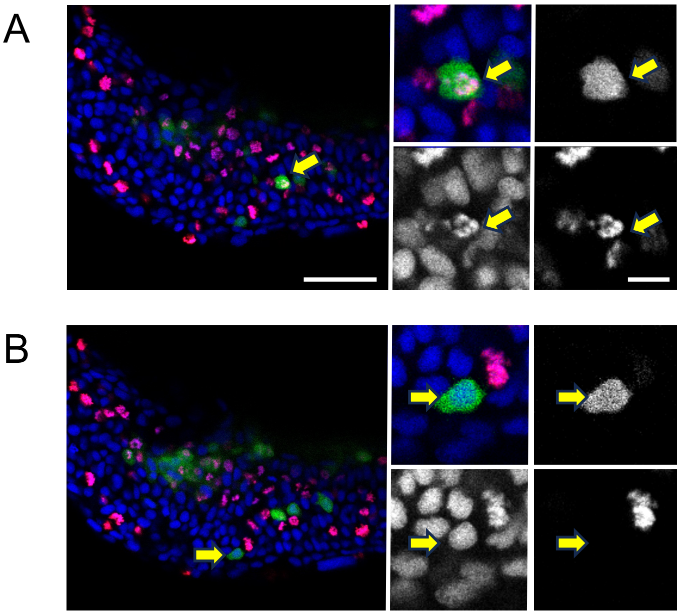

Supplementary Figure 4. Representative images of *mastl*^{kt441b} homozygous embryos stained with pHH3 (related to Figure 5G) A pHH3-positive detached cell with condensed chromosomes (A) and a pHH3-negative detached cell with decondensed chromosomes (B) are shown. Fixed *mastl*^{kt441b} homozygous embryos at the 12-somite stage were stained with anti-phosphorylated Histone H3 (pHH3) antibody (red) and DAPI (blue). Because analyzed embryos carrying the *Bac(foxa2:Kaede)* transgene*,* CNH cells were labeled with Kaede fluorescence (green). Sagittal planes of the tailbud are shown in the left panel (scale bar, 50 µm) and magnified images of cells indicated by yellow arrows are shown in the right panels (scale bar, 10 µm). Clockwise from upper left, merged, Kaede fluorescence, pHH3, and DAPI images are shown.

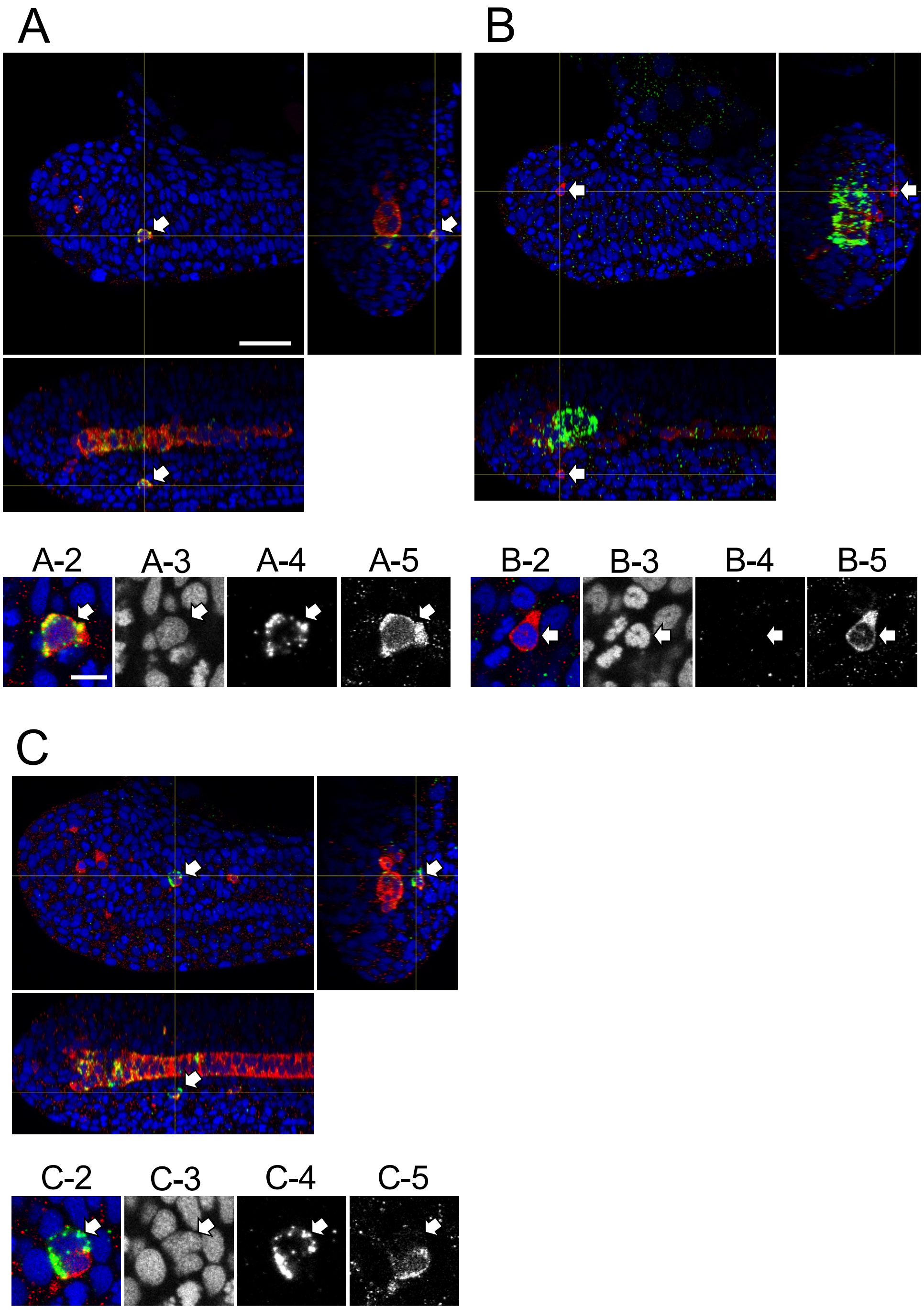

Supplementary Figure 5. Representative images of *mastl*^{kt441b} homozygous embryos stained with *foxa2* mRNA and Kaede (related to Figure 6A) Fixed *mastl*^{kt441b} homozygous embryos harboring the *Bac(foxa2:Kade)* transgene at the 16-somite stage were subjected to double staining with *foxa2* mRNA (green) and Kaede (red), and counter-stained with DAPI (blue). Tailbud regions were imaged sagittally and orthogonal views are shown. Images focusing on *foxa2* and Kaede double-positive cell (A), Kaede single-positive cell (B), and *foxa2* single-positive cell (C) are indicated (white arrows). Scale bar, 50 μm. Magnified images of cells indicated by white arrows in A-C are shown in the lower panels; merged (A-2, B-2, C-2) , DAPI (A-3, B-3, C-3), *foxa2* mRNA (A-4, B-4, C-4), and Kaede (A-5, B-5, C-5) are indicated. Scale bar, 10 μm.
